# Stroke management during the coronavirus disease 2019 (COVID-19) pandemic: experience from three regions of the north east of Italy (Veneto, Friuli-Venezia-Giulia, Trentino-Alto-Adige)

**DOI:** 10.1007/s10072-021-05066-9

**Published:** 2021-03-04

**Authors:** Paolo Manganotti, Marcello Naccarato, Ilario Scali, Manuel Cappellari, Bruno Bonetti, Alessandro Burlina, Emanuele Turinese, Sabrina Bogo, Francesco Teatini, Enrica Franchini, Giorgio Caneve, Giampietro Ruzza, Anna Gaudenzi, Roberto Bombardi, Giulio Bozzato, Roberta Padoan, Carolina Gentile, Michele Rana, Michelangelo Turazzini, Danese Alessandra, Francesco Brigo, Raffaele Nardone, Rocco Quatrale, Elisabetta Menegazzo, Maela Masato, Stefano Novello, Paolo Passadore, Antonio Baldi, Luca Valentinis, Claudio Baracchini, Alessio Pieroni, Anna Maria Basile, Claudio Semplicini, Silvio Piffer, Bruno Giometto, Simone Tonello, Domenico Marco Bonifatti, Simone Lorenzut, Giovanni Merlino, Maria Rosaria Valente, Francesco Paladin, Agnese Tonon, Cristina de Luca, Francesco Perini, Sandro Centonze, Paolo Bovi

**Affiliations:** 1 Stroke Unit, Clinical Unit of Neurology, Department of Medicine, Surgery and Health Sciences, Cattinara University Hospital ASUGI, University of Trieste, Strada di Fiume, 447, 34149 Trieste, Italy; 2 Stroke Unit, Azienda Ospedaliera Universitaria Integrata, Verona, Italy; 3 Stroke Unit, Ospedale San Bassiano, Bassano del Grappa, Italy; 4 Stroke Unit, Ospedale San Martino, Belluno, Italy; 5 Stroke Unit, Ospedale Centrale, Bolzano, Italy; 6 Stroke Unit, Ospedale di Cittadella, Cittadella, Italy; 7 Stroke Unit, Ospedale Santa Maria dei Battuti, Conegliano, Italy; 8 Stroke Unit, Ospedale Santa Maria del Prato, Feltre, Italy; 9 Stroke Unit, Ospedale di Gorizia, Gorizia, Italy; 10 Stroke Unit, Ospedale Mater Salutis, Legnago, Italy; 11 Stroke Unit, Ospedale “Franz Tappeiner”, Merano, Italy; 12 Stroke Unit, Ospedale dell’Angelo, Mestre, Italy; 13 Stroke Unit, Ospedale di Mirano, Mirano, Italy; 14 Stroke Unit, Ospedale Santa Maria degli Angeli, Pordenone, Italy; 15 Stroke Unit, Ospedale Di Portogruaro, Portogruaro, Italy; 16 Stroke Unit, Azienda Ospedaliera Università di Padova, Padova, Italy; 17 Stroke Unit, Ospedale Sant’Antonio, Padova, Italy; 18 Stroke Unit, Ospedale Santa Chiara, Trento, Italy; 19 Stroke Unit, Ospedale Ca’ Foncello, Treviso, Italy; 20 Stroke Unit, Azienda Sanitaria Universitaria Integrata, Udine, Italy; 21 Stroke Unit, Ospedale Santi Giovanni e Paolo, Venezia, Italy; 22 Stroke Unit, Ospedale San Bortolo, Vicenza, Italy; 23 Struttura Complessa Ricerca, innovazione clinico-assistenziale, qualità, accreditamento e rischio clinico, ASUGI, Trieste, Italy

**Keywords:** Stroke, COVID-19, Thrombolysis, Thrombectomy, Italy

## Abstract

**Background:**

Efficiency of care chain response and hospital reactivity were and are challenged for stroke acute care management during the pandemic period of coronavirus disease 2019 (COVID-19) in North-Eastern Italy (Veneto, Friuli-Venezia-Giulia, Trentino-Alto-Adige), counting 7,193,880 inhabitants (ISTAT), with consequences in acute treatment for patients with ischemic stroke.

**Methods:**

We conducted a retrospective data collection of patients admitted to stroke units eventually treated with thrombolysis and thrombectomy, ranging from January to May 2020 from the beginning to the end of the main first pandemic period of COVID-19 in Italy. The primary endpoint was the number of patients arriving to these stroke units, and secondary endpoints were the number of thrombolysis and/or thrombectomy. Chi-square analysis was used on all patients; furthermore, patients were divided into two cohorts (pre-lockdown and lockdown periods) and the Kruskal-Wallis test was used to test differences on admission and reperfusive therapies.

**Results:**

In total, 2536 patients were included in 22 centers. There was a significant decrease of admissions in April compared to January. Furthermore, we observed a significant decrease of thrombectomy during the lockdown period, while thrombolysis rate was unaffected in the same interval across all centers.

**Conclusions:**

Our study confirmed a decrease in admission rate of stroke patients in a large area of northern Italy during the lockdown period, especially during the first dramatic phase. Overall, there was no decrease in thrombolysis rate, confirming an effect of emergency care system for stroke patients. Instead, the significant decrease in thrombectomy rate during lockdown addresses some considerations of local and regional stroke networks during COVID-19 pandemic evolution.

## Introduction

In these months, the spread of SARS-CoV-2 disease (COVID-19) has reached pandemic proportions, affecting millions of people worldwide. The World Stroke Organization (WSO) has been monitoring experiences across the globe [1]. During the early COVID-19 emergency pandemic, in different countries, there was a significant service reorganization of most acute stroke services. Many healthcare systems reduced provision of “non-urgent” care, with a particular impact on stroke prevention, follow-up, and even urgent interventions such as thrombolysis and carotid endarterectomy. The ability to offer endovascular treatments has been reduced in many units [2, 3]. Beginning in mid-February 2020, the Italian authorities had to manage a huge reorganization to direct resources towards COVID-19 patient care [4]. At the end of May, Italy counted 227,364 diagnosed COVID-19 infections and 32,330 deaths (14.2%) (ISS). The rapid surge of COVID-19 cases seriously threatened the Italian health system: many regions such as Lombardia (the largest in Italy with >10 million inhabitants, but also the one with the highest number of COVID-19 cases) decided to dedicate entire hospitals to the increasing need of COVID-19-positive patients. In contrast, the care of other non-COVID-19 diseases as well as non-urgent interventions and outpatient activities was highly reduced or even stopped [2, 3, 5]. Acute stroke pathways were also completely redrawn: pre-hospital transportations were reorganized to prioritize the SARS-CoV-2-infected patients’ needs; specific triage protocols were activated to assess and manage COVID-19 suspicion or infection. Many stroke units were closed to reallocate stroke physicians and nurses to the care of COVID-19 patients or even converted into COVID-19-positive wards [6].

Recently, different studies reported the experience of stroke management during the COVID-19 period to guarantee the continuity of care and emergency [7–11]. A lot of multicentric studies were done in many countries (the USA [12–15], China [16], the UK [17], France [18], Spain [19, 20], Singapore [21], Germany [22, 23], Denmark [24], and Holland [25]), but in Italy, we had only single-center studies [26, 27] before the comprehensive Italian study recently printed [11]. The principal aim of our work is to provide a snapshot of the North-Eastern part of Italy stroke management during the early COVID-19 pandemic, focusing on few but clear items of emergency care, in a region counting 7,193,880 inhabitants based on the last demographic data (ISTAT).

In this study, we reported the data of admission of ischemic stroke patients, the number of thrombolysis and the number of thrombectomy in 22 stroke units in Triveneto (i.e., Friuli-Venezia-Giulia, Veneto, and Trentino-Alto-Adige regions) during the pre-lockdown and lockdown periods documenting the medical system emergency activity in stroke management.

## Methods

The retrospective data acquisition period ranged from January 2020 to May 31, 2020, and was performed at 22 stroke centers of the three regions (12 comprehensive stroke centers and 10 primary stroke centers). Numbers of patients admitted with final diagnoses of ischemic stroke from January 1, 2020, to May 31 and information regarding reperfusive therapies (intravenous thrombolysis and mechanical thrombectomy) were gathered from main discharge diagnosis documentation according to the local hospital and Italian diagnosis-related group system and SPREAD-ISO criteria. Treatment was defined by Operation and Procedure Classification System codes for intravenous thrombolysis and thrombectomy. All patients with acute stroke receiving treatments were included.

This survey was conducted in line with the principles of the Declaration of Helsinki. Approval for the study was obtained from the local ethics committee (CEUR FVG).

### Endpoints

The primary endpoint was the total number of ischemic stroke patients admitted to the stroke units. Secondary endpoints were the number of patients receiving thrombolysis and the number of patients receiving the thrombectomy.

### Statistics

To test the impact of the outbreak on the COVID-19 pandemic on stroke patients’ volume and patients’ characteristics, we analyzed the data in the following ways. The temporal distribution of admissions, thrombolysis, and thrombectomies was tested for homogeneity with a chi-square goodness of fit; if differences were significant, we used multiple comparisons Friedman rank test (*p*<0.05 considered significant). Moreover, patients were divided into two groups (pre-lockdown and lockdown periods) and differences on the number of admissions, thrombolysis, and thrombectomy procedures were tested using the Kruskal-Wallis test (*p*<0.05 considered significant). All the analyses were performed using SPSS Statistics 23 (IBM, Armonk, NY, USA).

## Results

We collected 2536 patients admitted to the 22 stroke units included in this survey, from January to May 2020. A reduction in admissions due to ischemic stroke was noted during the COVID-19 period using chi-square goodness of fit analysis, from a maximum in January (570, pre-COVID) and a minimum detected in April (464), reaching statistical significance on the Friedman rank test (*p*=0.016).

Focusing on management and treatment, thrombolysis was performed during the non-COVID-19 and COVID-19 period without differences in all Triveneto stroke units: a total of 609 alteplase infusions were almost equally divided into the 5 months examined, with a variable trend express in the single centers. The percentage of thrombolysis on admission is almost unvaried in COVID-19 months (March and April). The chi-square goodness of fit analysis did not reveal temporal distribution inhomogeneities.

Endovascular treatment showed a significant reduction during these months (both in absolute values and in percentage of admissions). Analyzing a total of 267 thrombectomies, a halving of numbers in April (32) compared to January (64) and a statistically significant inhomogeneity of data distribution using chi-square goodness of fit analysis can be seen, confirmed by the Friedman rank test (*p*=0.032): the majority of Triveneto centers individually confirm this trend. Categorizing data into two groups (non-COVID-19 period—January and February—and COVID-19 period—March and April) and analyzing these with the Kruskal-Wallis test, there was a significant decrease of admissions (11%) with a collapse of thrombectomies (42%), while the rate of thrombolysis was uniform (*p*=0.025).

**Fig. 1:**
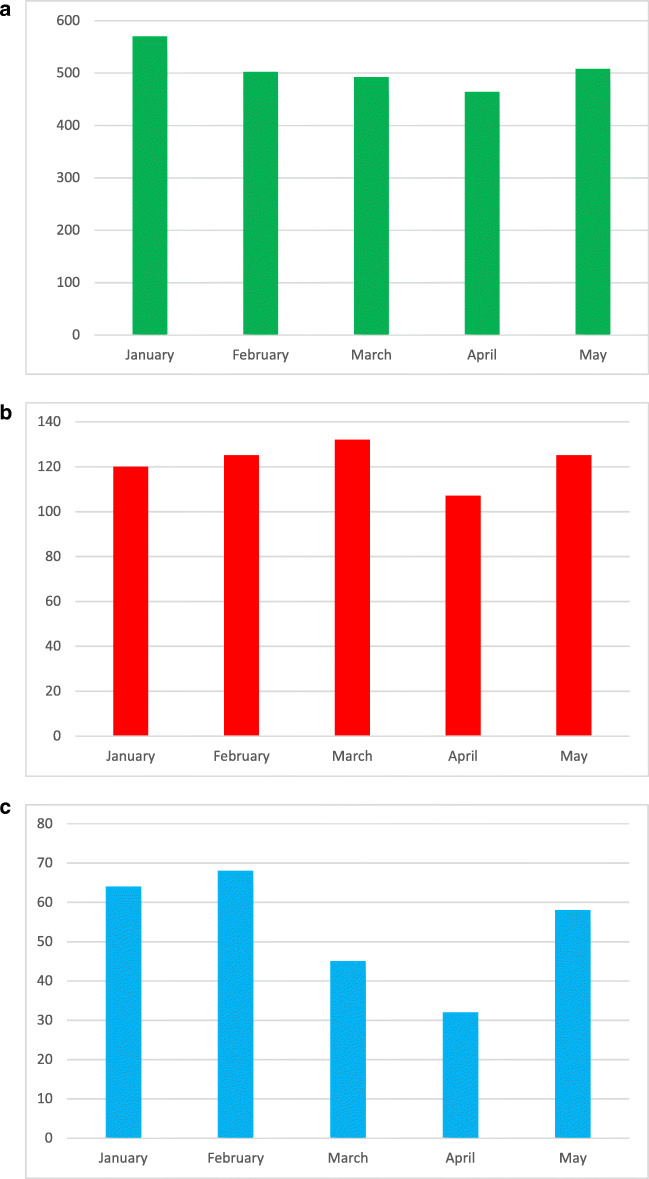
Number of patients admitted to the Northern-East Italy stroke units (**a**) during non-COVID-19 and COVID-19 periods (January to May 2020). Number of thrombolysis (**b**) and endovascular treatments (**c**) done during the same period in these stroke units

**Fig. 2:**
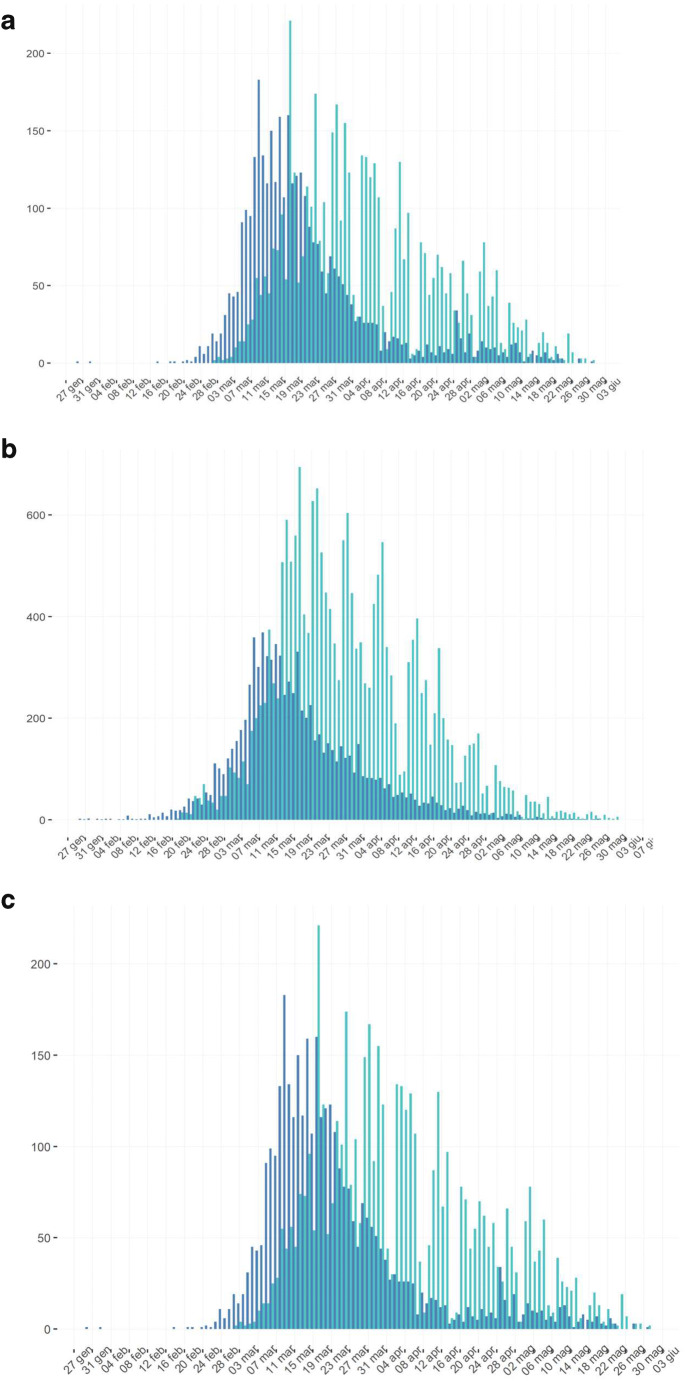
Trend of COVID-19 symptoms (dark blue) and diagnoses (light blue) in Friuli-Venezia-Giulia (**a**), Veneto (**b**), and Trentino-Alto-Adige (**c**) from January to May 2020. http://www.dati.salute.gov.it, accessed June 05, 2020

None but one of the stroke units of the three regions was reorganized in COVID-19 units or redrawn in emergency units. There was no dedicated stroke unit for COVID-19 patients: every patient with acute ischemic stroke was treated regardless of his positivity to the coronavirus (Figs. 1 and 2).

## Discussion

The first main finding of our survey is a reduction of admissions of ischemic stroke patients in dedicated stroke units during the lockdown period (especially in April 2020) in a large region of Italy, counting 7,193,880 inhabitants. This result is in line with some published reports [11, 28, 29], but extends the previous observations to a different area. Most centers in our report participated also to the Italian survey [11] but the results cannot be directly compared, because the two studies have different time courses, endpoints, and statistical analyses.

The second main finding of our survey is that absolute and relative number of thrombolysis was constant across all included stroke units: none of the stroke units involved reported significant decrease of thrombolysis treatment in the different regions of the North-East area of Italy. In contrast to our findings, another study recently printed [11] reported a decrease of thrombolysis during March 2020 in other Italian regions. We could just consider few suggestions to explain these data. Across the stroke units, there was an organized filtering and protection for suspected COVID-19 patients with stroke that were undertaken [2, 3] during the COVID-19 pandemic. Dedicated protected rooms in stroke units for stroke patients’ emergencies requiring fast reperfusive therapies before result of COVID-19 swab tests, specific training for operators, and quick swab tests for each new stroke patient probably did not affect the possibility of reperfusive therapies in comprehensive stroke centers as well as in primary stroke centers. We shall underline the sensibility of emergency system to the stroke networks well developed in all the territory of the north east and the presence of general practitioners alerting the emergency system in case of stroke patients. Finally, to explain the persistence of thrombolysis without a decrease of treatment as described in an Italian study [11], we should consider that the data presented in our paper are collected from January to May 2020. We focused more on an admission and treatment temporal trend than on a comparison between 2020 and 2019: our primary aim was to describe the effect of whole COVID-19 first pandemic wave on stroke emergency. Furthermore, our findings were obtained from areas with similar diffusion of pandemic and similar approach to health emergency.

To explain the discrepancy between reduction of admission and persistence of reperfusion therapy before and during the COVID-19 period, we noted that minor stroke patients did not arrive to emergency care or probably were treated at home by general practitioners and this finding is in line with the Italian national survey [11]; in contrast, “severe” or “significant” stroke patients were addressed to the hospital even in the more severe period of the COVID-19 pandemic. These findings across different centers guarantee the continuity of care of stroke patients in the dramatic COVID-19 pandemic in these three regions. The finding underlies the reactivity of the stroke emergency system similar to other European reports (as in Germany [22, 23]).

In contrast to the persistence of intravenous thrombolysis, the other dramatic finding of our study was a significant decrease of thrombectomies during the lockdown period, mainly in April 2020 with a recovery in May 2020 across all the stroke units. This oscillation follows the peak and the descending slope of pandemic as reported by ISTAT. Similar patterns of performance are reported in a multicenter French study [7] describing a significant decrease in stroke patients treated with thrombectomy during the first stages of the COVID-19 pandemic in France. Actually, we can only speculate on the different causes responsible for this finding. We can consider a delay of time due to the emergency period, but for these patients we did not collect the time from the onset of sympotms to the arrive in emergency department. In our opinion, possible causes could be the major difficulty of the interventional radiology units connected to the stroke units to perform filtering and dedicated room, the reduced number of dedicated operators (which were involved in other radiological activities with risk of contamination), the difficulty of rapid sanification of the interventional rooms, and mostly a reduction of acute stroke patients transport from primary stroke centers to comprehensive stroke centers, in line with the French survey [7]. This finding however was transitory for the month of April and recovered to the previous level in May suggesting the rapid reactivity also of this system in the three regions contemplated.

Our findings on thrombectomy are in contrast with the Italian national survey [11] but in line with the French one [7] that considered almost the same timeline as the Italian survey. In Northern Italy (North-West and probably Emilia-Romagna), a mothership transport approach to comprehensive stroke centers was predominant, since many primary stroke centers were converted to COVID-19-treating units; this could explain the increase of thrombectomies as well as the decrease of thrombolysis in those areas. In our region, the stroke network remained almost unchanged during the pandemic, with both primary stroke centers and comprehensive stroke centers dealing with ischemic strokes. The different organization of health services with other Italian areas with the same COVID-19 incidence could explain the different results in terms of thrombolysis and thrombectomies. Furthermore, our survey just considered the pandemic (March, April, and May) and pre-pandemic (January, February) periods, so monthly variance in ischemic stroke incidence and reperfusion rate could be a confound factor.

These findings prompt immediate consideration of local and regional stroke networks preparedness in the varying contexts of COVID-19 pandemic evolution. Fear of in-hospital infection and advices from health authorities, media, and doctors probably led patients with mild stroke symptoms to stay at home and to be treated by general practitioners. For this reason, recent information campaigns, encouraging patients to an early emergency room presentation, were increasingly implemented from stroke physicians.

The reactivity of the stroke network was similar in all the three regions of the North-East of Italy, which have similar organization in comparison to other regional lands of Italy. This report underlies the organizations of the system during this dramatic pandemic and the information here reported could be useful for other systems and for future ongoing peaks of pandemic in other countries. Based on these data, in our opinion, quick swab test, filtering and dedicated rooms and operators in stroke units, sensitivity for emergency, and connection with general practitioners were probably the reasons of preservation of emergency treatment of stroke in North-East of Italy despite the COVID-19 pandemic.
